# Book Review: Addictions From an Attachment Perspective: Do Broken Bonds and Early Trauma Lead to Addictive Behavior?

**DOI:** 10.3389/fpsyg.2019.02170

**Published:** 2019-10-04

**Authors:** Katelyn Rinker

**Affiliations:** Department of Psychology, Washington State University, Pullman, WA, United States

**Keywords:** addiction, alcoholism, self-harm, psychotherapy, self-medication

The development of addiction through poor attachments during childhood is a crucial area of research. John Bowlby, who created the Attachment Theory, inspired Richard Gill to gather together relevant scholarly papers in his honor (Gill, 2017). These unique papers formed a book known as *Addictions from an Attachment Perspective*, which is targeted for a broad audience of clinical psychologists and psychoanalysts. Richard Gill works at St. Joseph's hospital while providing treatment programs for various addictive behaviors in the United Kingdom. He is the director of London's Action on Addiction program, which has helped serve individuals who struggle with substance use disorders for half a decade.

John Bowlby emphasized that healthy attachments are needed “from the cradle to the grave” (Gill, 2017). Bowlby conducted research in psychoanalysis, which is how he discovered his theory on attachment in the early 1900s. He enlightened the world with his theories on the relation between addiction and negative emotions, such as loss and deprivation (Gill, 2017). Bowlby started his extraordinary work with orphaned children who had lost their parents through war (Gill, 2017). Bowlby is one of the best minds of the 20th century to discover the clear relationship between traumatic experience and human development (Gill, 2017).

The book covers attachment's relation to personal identity, gender, and culture. The goal of this book, in the exact words of the author, is to provide an “in-depth understanding that addictions are a response to, and hold the pain of, broken attachments and are best treated within healthy interpersonal relationships” (Gill, 2017). A quick summary of the book includes the topics of treating addiction, coping with self-medication, and using alcohol (Gill, 2017). Other topics also arise, such as avoiding self-harm, abstaining from drugs, and seeking help with gambling addictions (Gill, 2017). The main theme throughout the pages is promoting healthy attachments and healing those with addictions.

The book jumps into the lively discussion of the Attachment Theory. A key notion promoted in the book explains that the reason that addiction occurs is to “provide the soothing and safety which are the features of an internalized secure base and from which the person can emerge and engage in exploration” (Gill, 2017). After all, it has been well-known for decades that many of the young adults or high school students who try drugs may claim that they are only “experimenting” with substances (Swadi, 1990). This theory acts as if their drug use is supposed to be a one-time occurrence, which ignores the fact that this special occasion can turn into a daily habit and possibly drug dependence. According to Omu et al. (2017), adolescents are the population who is most likely to use drugs. So health concerns are raised when looking at young populations and their risk of drug use.

*Addictions from an Attachment Perspective* claims that broken attachments, such as the loss of a loved one in the time of war, may lead to addiction (Gill, 2017). Edward Khantizan was the first to mention this theory of the “Self-Medication hypothesis” when witnessed soldiers using drugs or alcohol to numb their pain (Gill, 2017). The soldiers' discomfort extended long past the battlefield as they brought their distress back home with them in the form of Post-traumatic Stress Disorder (Gill, 2017). This psychiatric condition forced the soldiers to relive their horrific experiences of extreme loss.

Self-medication can be a reason for alcoholism. Chronic alcohol use can negatively impact attachments by altering the roles of caregiving, affection, and comfort in relationships (Gill, 2017). These relationships may be romantic interferences or family obligations. The book by Gill states that heavy alcohol use affects attachments because the individual begins to “rely on alcohol before people and to trust alcohol to “look after them” more reliably than any person could or might” (Gill, 2017). Alcoholic behavior replaces the need for love or belonging, which previously had acted as a motivation to find new attachments or build current ones. The individual who struggles with alcohol addiction distances themselves off from emotional attachments. This lack of emotion creates an “absent or dead” relationship between husband and wife, or mother and child (Gill, 2017). Or apathy can end relationships and break attachments due to this cold perspective.

Psychotherapy can be helpful for allowing those with alcoholism to connect with their spouse or family (Gill, 2017). But this treatment needs to include exploration of emotion and engagement in the curiosity behind their personal relationships (Gill, 2017). It also requires a heightened sense of empathy, along with the willingness to seek out the emotional experiences of their partner or family member (Gill, 2017). And lastly, the psychotherapy session needs a calm atmosphere (Gill, 2017). A peaceful environment may allow for the focus of treatment to be on forming healthy relationships that can be used instead of alcohol.

Other types of addiction can include the overuse of technology, gambling, or promiscuous behaviors (Gill, 2017). Hypersexuality is a problematic behavior that relies on attachments *too much*, which is the same as excessive technology use through social media (Gill, 2017). Researchers who published in the *Clinical Neuropsychiatry* journal suggest that gambling involves lack of self-control that is similar to a drug addiction (Terrone et al., 2018). Impulsivity is especially high among individuals with substance abuse disorder.

Bowlby's theories paved the road for new psychoanalysts who would later contribute to the vast fields of developmental psychology, sociology, and psychoanalysis. Research that is inspired by Bowlby includes studies on attachment and addictive behavior. These studies promote the theory that inadequate childhood attachments, which develop from neglect or lack of parent-child bonding, may lead to drug dependence (Musetti et al., 2016). One study, published by *Frontiers in Psychology*, looked at 57 participants with poor attachments during childhood, and 47 of them were prone to drug use (Musetti et al., 2016). Drug use takes the place of a true relationship. Further research on attachment and addiction is encouraged to shed light on the importance of relationships during human development.

## Author Contributions

The author confirms being the sole contributor of this work and has approved it for publication.

### Conflict of Interest

The author declares that the research was conducted in the absence of any commercial or financial relationships that could be construed as a potential conflict of interest.
